# Psychometric Soundness of an Arabic Version of the Jefferson Scale of Attitude toward Physician and Nurse Collaboration (JSAPNC): A Preliminary Study

**Published:** 2017-05

**Authors:** Aymen ELSOUS, Ali AKBARI SARI, Mahmoud RADWAN, Samah MOHSEN, Hatem ABU ZAYDEH

**Affiliations:** 1. Dept. of Health Management and Economics, School of Public Health, International Campus, Tehran University of Medical Sciences, Tehran, Iran; 2. Quality Improvement and Infection Control Office, Shifa Medical Complex, Ministry of Health, Gaza Strip, Palestine; 3. Al Mustaqbal Research Center, Gaza Strip, Palestine

**Keywords:** Reliability, Validity, Psychometrics, Physician-nurse collaboration, Teamwork, Inter professional collaboration

## Abstract

**Background:**

The Jefferson Scale of Attitude toward Physician-Nurse Collaboration (JSAPNC) has been used to measure the attitude regarding collaboration between nurses and physicians. The aim of this preliminary study was to test the reliability and validity of an Arabic version of the questionnaire and adapt it for use in Palestine.

**Methods:**

Forward-backward translation of guidelines provided in the literature was followed. Content validity was examined by nine health experts and reliability was assessed with Cronbach’s coefficient alpha; test-retest reliability. Construct validity was explored with exploratory factor analysis (EFA) and confirmatory factor analysis (CFA) by means of survey among 414 physicians and nurses worked at Shifa Medical Complex in the Gaza Strip in 2015.

**Results:**

Response rate was 65% and Cronbach’s coefficient alpha was 73.2 for the entire sample. Test-retest reliability was 0.79 measured by Pearson correlation. Factor analysis with Varimax rotation revealed four factors explained 60.5% of the variance in the responses labeled as physician-nurse collaboration, doctor’s authority, Shared education and Nursing role in-patient care. Goodness of fit indices from the CFA showed a satisfactory model of fit; Comparative Fit Index (CFI) = 0.89; Root Mean Square Error of Approximation (RMSEA) = 0.06; Standardized Root Mean Square Residual (SRMR) = 0.03; and Hoelter index = 206.

**Conclusion:**

The Arabic version of JSAPNC is psychometrically sound tool with satisfactory measurement characteristics including validity and internal consistency reliability. Future research is required to replicate these findings with larger and representative sample. Generalization to Arab speaking countries can be considered but with caution.

## Introduction

Inter-professional collaboration is critical for patient care and outcomes. Teamwork and collaboration between physicians and nurses have seen as a rock component of professionalism because they have been found to be matched with improvement of health outcomes and quality of patient care (1) including reduction of mortality rate in inpatient settings (2), job satisfaction (3), maintaining patient safety (4) and lower health care cost (5).

The collaboration between physicians and nurses is a communicative process within the provision of patient care and is involving share of responsibility, solving common field problems, integration of patient care management and decision making through obvious and defective communication path (6). Collaboration means “collective action toward a common goal in spirit of trust and harmony” (7).

Teamwork and professional communication were seen as an important element of patient care, but many studies reported on the problems and complications of ineffective collaboration and teamwork for instance; medical errors, staff turnover and dissatisfaction. The joint commission’s national patient safety 2008 revealed that more than 60% of medical errors are due to ineffective communication and collaboration among care providers (8).

Various methodologies have been used to examine the collaboration between nurses and physicians including survey, focus group discussion and/or observation. The JSAPNC has been widely used in determining collaboration between both nurses and physicians (9, 10).

Nurses and physicians are the most people responsible for patient care, but they do not communicate properly (11). Differences in the attitudes of nurses and physicians toward collaboration were reported (12). Nurses perceived more collaboration than physicians perceive and had more positive attitude toward collaboration in inpatient settings (13). Findings from meta-analysis of 18782 professionals showed physicians, manifesting with greater power, but were less likely to express collaboration and sharing powers with nurses (13).

We aimed to assess the attitude toward collaboration between physicians and nurses in Palestinian hospitals using the JSAPNC, but the instrument is not ready and culturally adapted to be used in the Arabic and Palestinian context. Hence, the objective of this study was to test the validity and reliability of an Arabic version of JSAPNC.

## Materials and Methods

### Instruments

In this study, the attitude toward collaboration was measured by the JSAPNC (9), but modified in 2003 (10). It had been validated as a research tool in western countries (12, 14). The instrument measures the attitude toward collaboration with fifteen questions divided into four domains including: 1) Shared education and collaboration (7 items), 2) Caring as opposed to curing (3 items), 3) Nurse’s autonomy (3 items), and 4) Physician’s authority (2 items). The JSAPNC measures items on 4 point Likert scale (1= strongly disagree, 2= disagree, 3= agree, 4= strongly agree). Items 14 and 15 are reversed coded because they reflect unfavorable attitude toward collaboration. The overall score is range from 15 to 60 theoretically and higher sum scores on the JSAPNC indicate positive attitudes toward physician-nurse collaboration.

### Forward and backward translation

The JSAPNC was handled by two independent bilingual health professionals for Arabic translation and emphasize during translation was focused on conceptual more than linguistic translation. Then researchers sat together and evaluated the quality of Arabic translation and inconsistency in translation was resolved by discussion between translators themselves. The word psychological counselling in item “Nurses have special expertise in patient education and psychological counseling” was easily translated. In return, we used psychological support instead of psychological counselling because registered nurses were not trained or educated enough to be counselors. The item “Physicians should be educated to establish collaborative relationships with nurses” was not typically translated because the physicians see themselves as perfect in establishing teamwork and collaboration with others especially the nurses. Moreover, culturally, the word “educated” cannot be used in this instance. Hence, the statement was modified to “Establishing collaborative relationship with nurses should be well considered by physicians”. The backward translation was done by another two independent translators never seen the original questionnaire before. Back-translation method is popular because it gives an indication of semantic equivalence and enhances the validity of the questionnaire.

### Face validity

To assess the face validity, the final Arabic version of JSAPNC was sent to 15 health professionals (nine nurses and six physicians). They were asked to assess the clarity, ambiguity and easy understanding of the JSAPNC items.

### Content validity

Content validity was examined through determining items and scale content validity index (I/CVI, S/CVI) using the average approach (CVI/Ave) (15). This approach estimated CVI as proportion of items that received a rating of 3 or 4 by the experts. The Arabic version was sent to 12 health professionals (five academics and seven health experts) and was asked to rate the relevancy of items using the 4 points Likert scale (1= not relevant, 2= quite relevant, 3= somewhat relevant, 4= highly relevant) (16). Rating of 1 and 2 was considered “content invalid”, while rating of 3 and 4 was considered “content valid”. Nine experts responded and content validity index was calculated accordingly.

### Construct Validity

Construct validity was tested by means of survey among 414 nurses and physicians by exploratory factor analysis (EFA) and confirmatory factor analysis (CFA). With CFA, we calculated the following indices to assess goodness of fit: Chi-square statistics (degrees of freedom [df], *P-*value) (17), CFI > 0.90 (18). Tucker Lewis Index (TLI > 0.90) (18), RMSEA ≤ 0.06 (18), Hoelter index at least 200 (18), SRMR < 0.08 (19), and its 90% confidence interval.

### Reliability

Reliability of the Arabic JSAPNC was assessed by test-retest reliability, determined by Pearson correlation, among 21 nurses and physicians with two weeks period gap. Test–retest reliability indicates the degree of score stability over time. Internal consistency was assessed by Cronbach’s coefficient alpha above 0.65 indicates that items measure the same concept.

### Psychometric testing of the JSATPNC/Arabic version based on survey data

The hospital based cross-sectional study took place in Shifa Medical Complex, the largest and oldest hospital in the Gaza Strip, Palestine, with 613 beds capacity. It locates in Gaza City and comprises three main hospitals: surgical, internal medicine and maternity hospital. It has 1373 employees among them 543 are nurses and 423 are physicians.

### Study population

All nurses and physicians who worked in Shifa Medical complex during study period and met the inclusion criteria:

- Be a formal employee: internship staff, trainee, and volunteer was excluded
- Has at least six months working experience
- Willing to participate in the study.

### Sampling and sample size

The total number of physicians and nurses who met the criteria for participation in the study was 219 and 418 respectively (census sample). The JSAPNC was distributed to a total of 637 physicians and nurses.

### Ethical approval

The Institutional Review Board of Shifa Medical Complex approved the study. Study’s objectives were explained to participants and informed consent was obtained orally. Enrollment in the study was voluntary based and participants were anonymous.

### Data collection

Data was collected during working hours in the morning shift by the researchers themselves for three consecutive months from October to December 2015, following face-to-face interview approach.

## Results

### Physicians and nurses characteristics

We invited 637 nurses and physicians to participate in the study, but 414 responded (response rate 65%). The response rate for nurses and physicians was 74.9% and 46.1%, respectively. Male to female ratio was 2:1 and mean age was 37.04 ± 9.95 yr (median = 34 yr), while mean years of experience was 11.66 ± 8.26 yr (median = 9 yr).

Majority of physicians were male (96%), had more than 35 yr old (80.2%) and half of them had experience at work between 11–20 yr (56.4%). Nurses are not alike, they were less than 35 yr old (62.9%) and had less than 10 yr experience at work (63.9%) (Table 1).

**Table 1:** Baseline characteristics of participants

	**Physicians (n=101) n (%)**	**Nurses (n=313) n (%)**	**Total (n=414) n (%)**
Gender			
Male	97 (96.0)	182 (58.1)	279 (67.4)
Female	4 (4.0)	131 (41.9)	135 (32.6)
Age			
≤ 35 yr	20 (19.8)	197 (62.9)	217 (52.4)
> 35 yr	81 (80.2)	116 (37.1)	197 (47.6)
Place of work			
Surgical		147 (47.0)	224 (54.1)
Internal medicine	77 (76.2)	81 (25.9)	105 (25.4)
Maternity	24 (23.8)	81 (25.9)	81 (19.6)
	-------		
Years of experience			
≤ 10 yr	33 (32.7)	200 (63.9)	233 (56.3)
11 – 20 yr	57 (56.4)	63 (20.1)	120 (29.0)
> 21 yr	11 (10.9)	49 (15.7)	60 (14.5)
Education			
Diploma	---------	117 (37.1)	117 (28.3)
Bachelor			191 (46.1)
Master	27 (26.7)	164 (52.4)	62 (15.0)
PhD	41 (40.6)	21 (6.7)	11 (2.7)
Board	7 (6.9)	4 (1.3)	25 (6.0)
	25 (24.8)	----------	

### Reliability assessment

The Cronbach’s coefficient alpha value for the 15-item JSAPNC was 73.2 (74.7 – 89.5) and test-retest reliability as measured by Pearson correlation was 0.79. Descriptive statistics including test-retest of the 15 items are reported in Table 2.

**Table 2:** Physician and Nurse test-retest reliability

	**Test (n = 21)**	**Retest (n = 21)**
Mean (SD)	47.95 (5.44)	45.17 (3.50)
Median	47.50	46.00
Mode	46.00	47.00
Minimum	41.00	39.00
Maximum	56.00	50.00
Range	15.00	11.00
Pearson correlation		0.79

### Content validity

Nine experts rated the relevance of the Arabic JSAPNC items and I-CVI and S-CVI have calculated accordingly. The I-CVI and S-CVI ranged from 0.77 to 1.00 and 0.86 to 0.94 respectively. The Kappa statistic (k*) for questionnaire’s items ranged from 0.76 to 1.00.

### Construct validity

**Fig. 1:**
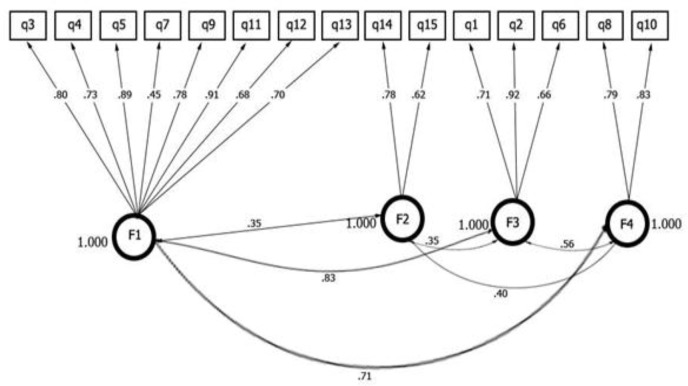
Confirmatory factor model-Jefferson Scale of Attitude toward Physician and Nurses Collaboration/ Arabic version

Confirmatory factor analysis was performed on the 15 items of the Arabic JCAPNC. The factor structure model is presented in Fig. 1.

Adjusting the final 15 items model was satisfactory and acceptable: Chi-square = 235.474, df = 84, CFI = .89, RMSEA = .06, SRMR = .03, TLI = .86, Hoelter index equal to 200 and Goodness of fit (GFI = .92).

Exploratory analysis was done by factor analysis with SPSS version 20. Bartlett’s sphericity test of the 15 items (χ2= 1459.425 df = 105, *P*<0.001) indicated that the inter-item correlations were sufficient. The Kaiser Meyer Olkin (KMO), measures the sampling adequacy, is suggested to be at least 0.600 and the results of this study revealed KMO = 0.809. Exploratory factor analysis was performed with Varimax rotation matrix extracted four factors that explained 60.59% of total response variance. The results of factor analysis resulted in a simple structure and labels attached to each factor were developed considering all significant factor loadings. Eight out of 15 items were loaded on one factor and all extracted factors had items load greater than 0.360. In our model, the items loaded in four factors labeled as factor 1: Physician/Nurse Collaboration (8 items); factor 2: Doctor’s autonomy (2 items); factor 3: Shared education (3 items) and factor 4: Nursing role in patient care (2 items) (Table 3).

**Table 3:** Factors extracted and rotated factor loadings

**Item No**	**Items**	**Four factors structure (Hojat et al., 2003)**	**Four factors structure (results of our study)**				**Corrected Item total correlation**
			**F1**	**F2**	**F3**	**F4**	
	Physician / Nurse collaboration						
Q.3	A nurse should be viewed as a collaborator and colleague with a physician rather than his/her assistant	S	.365	.244	.349	.175	.487
Q.5	Physicians should be educated to establish collaborative relationships with nurses (Building collaborative relationship should be concerned by physician)	S	.558	−.038	.292	−.005	.562
Q.4	There are many overlapping areas of responsibility between physicians and nurses.	S	.685	.057	.052	−.021	.394
Q.7	Nurses should also have responsibility for monitoring the effects of medical treatment	S	.459	.049	.393	.180	.518
Q.12	Nurses should be involved in making policy decisions concerning the hospital support services upon which their work depends	C	.656	.266	.063	.189	.676
Q.11	Nurses should clarify a physician’s order when they feel that it might have the potential for detrimental effects on the patient	N	.475	.238	.155	.131	.533
Q.9	Nurses should be involved in making policy decisions affecting their working conditions	N	.538	.206	.010	.217	.566
Q.13	Nurses should be accountable to patients for the nursing care they provide	N	.652	−.061	.138	.041	.595
	Doctor’s authority						
Q.14	The primary function of the nurse is to carry out the physician’s orders	D	.120	.858	.028	.000	.813
Q.15	Doctors should be the dominant authority in all health care matters	D	.139	.907	.043	.065	.837
	Shared education						
Q.6	Physicians and nurses should contribute to decisions regarding the hospital discharge of patients	S	.164	.401	.689	.307	.587
Q.2	Interprofessional relationships between physicians and nurses should be included in their educational programs	S	.239	−.128	.504	.073	.550
Q.1	During their education, medical and nursing students should be involved in teamwork in order to understand their respective roles	S	.036	.147	.798	−.023	.674
	Nursing role in patient care						
Q.10	Nurses have special expertise in patient education and psychological counseling (Nurses have special expertise in patient education and psychological support)	C	.135	.073	.057	.839	.794
Q.8	Nurses are qualified to assess and respond to psychological aspects of patients’ needs	C	.099	.028	.101	.886	.811

**S:** Shared education and collaboration (items 1–7); **C:** Caring vs curing (items 8,10,12); **N:** Nurse’s autonomy (items 9,11,13); **D:** Doctors authority (items 14,15).

**F1:** Physician / Nurse Collaboration (items 3,4,5,7,9,11,12,13), **F2:** Doctor’s authority (items 14,15), **F3:** Shared education (items 1,2,6), **F4:** Nursing role in patient care (items 8,10)

## Discussion

This study provides important findings of psychometric properties of the Arabic version of the JSAPNC. Our response rate (65%) was lower to the response rates of previous JSAPNC administrations in other countries (20–22) but better than two studies (23, 24).

Findings from EFA suggested a four factors structure of attitudes toward physician-nurse collaboration fit to Hojat et al., (9), but unlike the 3 factors structure of ward et al., (25). In the factor analysis, the first factor, “physician–nurse collaboration”, eight items had load greater than 0.35 accounting for 28.9% of the variance. This factor shows the importance and the significant role of nurses and physicians in issues of decision-making and authority during the course of patient care and the role that nurses should play with physicians in determining the working climate. Four out of these eight items were loaded in its first factor (shared education and collaboration), one in its second factor (Caring as opposed to curing) and three in its third factor (Nurses’ autonomy) (9).

Two items loaded greater than 0.85 on our second factor and accounting for 14.4% of the variance, Doctors’ authority. This is similar to Hojat et al., (9) and Ward et al., (25) loaded in its fourth and third factor respectively and labeled as “Physician’s authority”.

Our third factor, shared education, contains three items loaded greater than 0.50 and accounting for 9.9% of the variance. These items were loaded in its first factor labeled as “shared education and collaboration” (9). The fourth factor, i.e., nursing role in patient care, had two items loaded greater than 0.83 and accounting 7.2% of the variance. These items loaded in its third factor labeled as “Caring as opposed to curing” (9).

This factor emphasizes the significant role of nurses in delivering patient care especially with the exclusive relationship between nurses and patients. This relationship is an advantage for nurses to cover the psychological aspects of health care.

The primary difference between our findings and the other study (9) is in the first bulky factor. In our study, items of the first factor were allocated in two factors: four in factor one and three in factor three. The second difference is the three items labeled as “Nursing autonomy” are loaded in our first factor labeled as “Physician / Nurse Collaboration”. The third difference is around two factors, which encompassed each two items. The differences could possibly be due to different sample population. The sample of Hojat et al. (9) was among first-year medical and nursing students, while ours was among professional nurses and physicians. The similarities are in the number of factors extracted and factor two, doctors’ authority, which addresses the physicians’ power during the course of treatment and patient care.

The reliability of an overall score and of subscales was adequate and acceptable, unlike findings in which reliability of subscales was not acceptable (23, 24). All item analysis suggested that corrected total item correlations were greater than desired value unlike some results (26). The statistically significant and positive item total score correlations (*P*<0.05) means that each item contributes to a significant degree to the total score of the scale.

Confirmatory factor analysis of the 15 items generally presented an acceptable and satisfactory model of fit. The *P*-value of less than 0.001 is the fit of the model to the data, TLI = 0.86 and CFI = 0.89 are below the recommended cut-off values. RMSEA = 0.06 equals the cutoff value of good model and SRMR = 0.03 is consistent with the recommended value of < 0.05.

Regarding content validity, the kappa statistic was above 0.75 for all items and according to previous parameters (27, 28); it indicates excellent and high agreement. Values of I-CVI and S-CVI indicated acceptable culture relevance (29) and 0.75 is the least recommended value for item indicating good content validity (30). For S-CVI, a value of 0.90 or above is recommended (28), while a value of 0.83 or more was suggested indicating good content validity and participants had no problem in understanding the questionnaire items (30).

This study was strengthened by including nurses and physicians from various disciplines. Limitations of this study include involvement of one hospital only and lower response rate, especially among physicians. We recommend replicating this study in future in a larger and a representative group.

## Conclusion

The Arabic JSAPNC is psychometrically sound tool for assessment of physician-nurse collaboration with a satisfactory to good measurement characteristics including goodness of fit structure and internal consistency reliability. The newly adapted instrument can be used with confidence to assess empirically the changes in attitude of nurses and physician toward collaboration in different settings and specialties.

## Ethical considerations

Ethical issues (Including plagiarism, informed consent, misconduct, data fabrication and/or falsification, double publication and/or submission, redundancy, etc.) have been completely observed by the authors.
